# Association of inflammatory markers and surgical intervention with postoperative pneumonia in patients with femoral intertrochanteric fracture: A propensity score-matched cohort study

**DOI:** 10.1016/j.jor.2025.06.025

**Published:** 2025-07-03

**Authors:** Fang Li, Xiaojun Fu, Yingding Ruan, Juncheng Yu, Junhua Chen, Liming Xu, Jie Xiao

**Affiliations:** a Department of Orthopedics, The First People's Hospital of Jiande, Jiande, China; b Department of Thoracic Surgery, The First People's Hospital of Jiande, Jiande, China

**Keywords:** Femoral intertrochanteric fracture, Inflammatory marker, Postoperative pneumonia, Proximal femoral nail antirotation, Surgery

## Abstract

**Background:**

This study was conducted to determine the value of inflammatory markers and surgical intervention for predicting the occurrence of postoperative pneumonia (POP) in patients undergoing proximal femoral nail antirotation (PFNA) surgery for femoral intertrochanteric fracture (FIF).

**Methods:**

A retrospective cohort analysis was conducted on patients with FIF who underwent PFNA surgery at The First People's Hospital of Jiande from January 2021 to December 2024. Systematically documented variables included preoperative and postoperative inflammatory biomarker levels, demographic characteristics, surgical approach and duration, and postoperative outcomes. The prognostic capacity of inflammatory markers for predicting POP was evaluated through a propensity score-matched comparative analysis framework.

**Results:**

Among 335 patients, 53 (15.8 %) had POP. After matching, 193 patients (POP group: n = 49; non-POP group: n = 144) were included in the analysis. The median (25th percentile, 75th percentile) postoperative systemic immune–inflammation index and neutrophil-to-lymphocyte ratio (NLR) were significantly higher in the POP group than in the non-POP group (1832.00 [1388.00, 3369.67] vs. 1261.76 [936.44, 1893.94], respectively P < 0.001, and 13.43 [10.85, 16.67] vs. 7.89 [5.32, 11.00], respectively, P < 0.001). Multivariate analysis showed that postoperative NLR was an independent predictor of POP (area under the receiver operating characteristic curve 0.8396, P < 0.001).

**Conclusion:**

Postoperative NLR may predict POP among patients undergoing PFNA surgery for FIF. However, the clinical utility and optimal thresholds require validation in future prospective studies.

## 1 Introduction

Hip fractures, particularly femoral intertrochanteric fractures (FIF), pose a significant public health challenge in older adults. These fractures account for 45 %–50 % of all hip fractures and have seen an increased incidence due to global aging. 1, 2, 3, 4 FIF, often termed the “last fracture of life,” primarily affects individuals aged >60. It is associated with high mortality rates, with 20 %–30 % of patients dying within one year post-surgery, 5, 6, 7, 8 substantial morbidity, reduced quality of life, and considerable economic burden. 9, 10, 11

Intramedullary nailing is the standard treatment for FIF, demonstrating favorable outcomes. ^4^^,^^12^ The Proximal Femoral Nail Antirotation (PFNA) system, which offers rotational and angular stability via a helical blade, has gained widespread use with evidence backing its efficacy. 13, 14, 15

Postoperative pneumonia (POP) occurs in 5.8 %–18 % of hip fracture surgery patients. 16, 17, 18, 19 It extends hospital stays, raises healthcare costs, and increases mortality risk. 20, 21, 22 Assessing inflammatory status may help predict POP risk in FIF surgery patients. While biomarkers like the systemic immune-inflammation index (SII) and neutrophil-to-lymphocyte ratio (NLR) have predicted pulmonary complications in other surgeries, ^23^^,^^24^ their role in orthopedic trauma is still underexplored.

To our knowledge, limited research has been done on the relationship between inflammatory biomarkers and POP in patients with FIF undergoing PFNA surgery. This study aims to explore this relationship and evaluate the potential of these biomarkers as predictors of POP through optimal threshold determination and propensity score matching (PSM).

## 2 Patients and methods

### 2.1 Study population and eligibility criteria

**Fig. 1:**
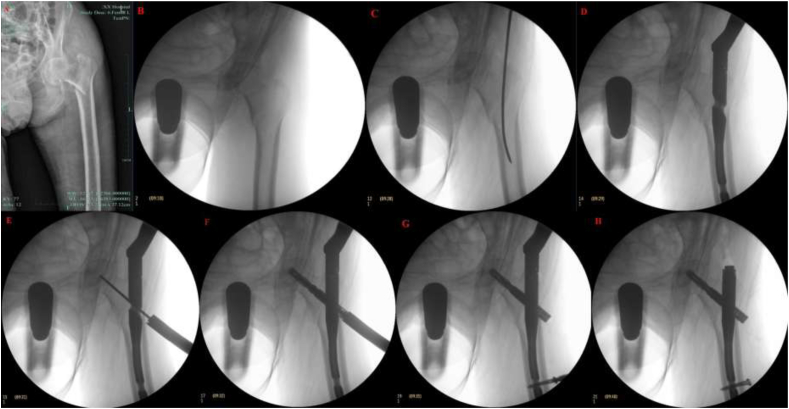
PFNA surgical procedure. A: FIF. B: Closed reduction was achieved through longitudinal traction. C: A guide pin was inserted laterally through the oval fossa. D: A 10 × 170 mm cephalomedullary nail was inserted through the medullary canal. E: A femoral neck guide pin was placed. F: A 90-mm helical blade was deployed into the femoral head. G: Distal fixation was achieved using a 36-mm locking screw. H: Fracture reduction. FIF, femoral intertrochanteric fracture; PFNA, proximal femoral nail antirotation.

The data of patients who underwent PFNA surgery for FIF (Fig. 1A) at The First People's Hospital of Jiande (Jiande, China) from January 2021 to December 2024 were retrospectively evaluated. The inclusion criterion was patients who underwent PFNA surgery for FIF. The exclusion criteria were patients (1) aged <18 years (n = 1); (2) participants transferred to alternative healthcare institutions (n = 1); (3) individuals with multiple surgical interventions within a 30-day period (n = 5); (4) patients presenting with infection or autoimmune disorders necessitating antibiotic or hormonal treatment, including Crohn's disease and systemic lupus erythematosus (n = 2); (5) with incomplete data (n = 4); and (6) with pulmonary infection at hospitalization (n = 4). Peripheral blood specimens were obtained from all participants within 72 h prior to surgery and 48 h postoperatively, with subsequent laboratory quantitation of inflammatory biomarkers.

The research was ethically implemented in strict adherence to the Declaration of Helsinki principles, with formal approval granted by the Institutional Ethics Committee of The First People's Hospital of Jiande (Ethics Committee Approval Number: 20250613-KY-001). In alignment with the study's retrospective methodology, the ethics committee formally dispensed with the requirement for written informed consent.

### 2.2 PFNA surgery

All patients underwent spinal anesthesia and were positioned supine on a radiolucent traction table. The affected limb was secured in traction-induced extension, with the contralateral limb in flexed abduction. Closed reduction was achieved through longitudinal traction, followed by fluoroscopic confirmation of anatomic alignment using a C-arm (Fig. 1B).

A 3-cm longitudinal incision was centered proximally over the greater trochanter, exposing the femoral head–neck junction. Under fluoroscopic guidance, a guide pin was inserted laterally through the oval fossa (Fig. 1C). The pin trajectory was verified radiographically, followed by sequential reaming of the greater intertrochanteric entry point. A 10 × 170 mm cephalomedullary nail was inserted through the medullary canal, and its depth and alignment were confirmed by the C-arm (Fig. 1D).

Using a targeting device, a femoral neck guide pin was placed under fluoroscopic control (Fig. 1E). After reaming, a 90-mm helical blade was deployed into the femoral head (Fig. 1F). Distal fixation was achieved using a 36-mm locking screw, which was inserted under fluoroscopic guidance (Fig. 1G). Final imaging demonstrated satisfactory fracture reduction, implant positioning, and hardware stability (Fig. 1H). After irrigation, the surgical incision was sutured. All components were provided by Waston Medical (Changzhou, China).

### 2.3 Data collection

The following data were retrospectively collected: demographic characteristics (sex, age, body mass index [BMI], and smoking history), clinicopathologic features, comorbidities (hypertension, diabetes mellitus, coronary heart disease, cerebral infarction, and chronic obstructive pulmonary disease [COPD]), Injury Severity Score (ISS), surgical approach, resection site, surgical duration, intraoperative blood loss volume, length of postoperative hospital stay, and incidence of POP.

The following inflammatory markers and other markers were measured to assess the preoperative and postoperative status: albumin, hemoglobin, NLR, PLR, LMR, and SII.

### 2.4 Observation indicators

This study evaluated two outcomes. The first outcome was the incidence of POP. POP was diagnosed based on the presence of at least three of the following features: (1) lung exudation and consolidation on chest radiography or computed tomography, (2) fever (body temperature of >38 °C), (3) white blood cell count of >10,000/mm^3^ or <3000/mm^3^, and (4) opportunistic pathogens in the sputum or bronchial secretions obtained by bronchoscopy. ^23^ The second outcome was the predictive value of preoperative and postoperative inflammatory markers for POP.

### 2.5 Statistical analysis

To enhance comparability and mitigate confounding bias, a 1:3 PSM cohort was generated using logistic regression modeling incorporating age, smoking status, BMI, and ISS. This methodology facilitated equitable distribution of baseline covariates across study groups. Post-matching equilibrium was evaluated via standardized mean differences (SMD), with established thresholds defining SMD <0.10 as negligible imbalance, 0.10–0.34 as minor, 0.35–0.64 as moderate, 0.65–1.19 as substantial, and ≥1.20 as critical imbalance. All covariates demonstrated SMD values within acceptable limits, validating cohort comparability.

Parametric continuous variables were analyzed using Student's t-test and reported as mean ± standard deviation (SD), while non-parametric data underwent Wilcoxon rank-sum testing and are presented as median [interquartile range (IQR)]. Categorical variables were compared through χ^2^ or Fisher's exact test, with results expressed as percentages. Binary logistic regression frameworks were applied for both univariate and multivariate analyses. Given the modest sample size post-matching (n = 193) with only 49 POP cases, we acknowledge the potential risk of overfitting. To mitigate this, we adopted a conservative variable selection approach, retaining only variables significant at p < 0.05 in univariate analyses and applying backward elimination (α = 0.05) to refine the multivariate model. The Hosmer-Lemeshow test confirmed adequate model calibration (p = 0.191), and the model demonstrated strong predictive performance (AUC = 0.8396). These results suggest that, despite the limited sample size, the model retains validity and generalizability.

Optimal threshold determination for preoperative inflammatory biomarkers was performed using receiver operating characteristic (ROC) curve analysis. Area under the curve (AUC) values ≥ 0.7 were considered clinically meaningful. All statistical tests were two-tailed with α = 0.05, and analyses were conducted in SPSS version 22.0 to ensure methodological rigor and precise characterization of variable relationships.

## 3 Results

### 3.1 Demographic and baseline characteristics

**Fig. 2:**
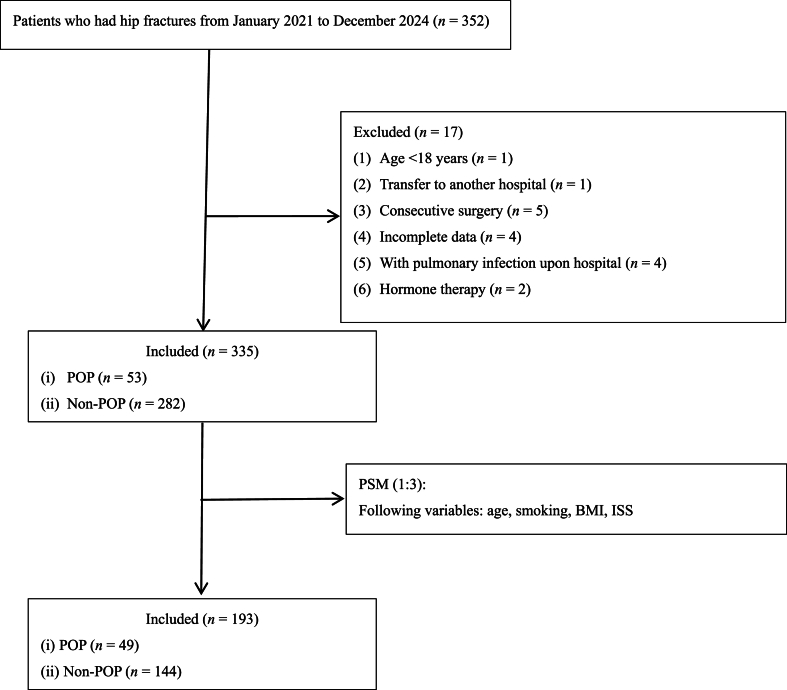
Flowchart of patient selection. BMI, body mass index; ISS, Injury Severity Score; POP, postoperative pneumonia; PSM, propensity score matching.

Overall, 352 patients who underwent surgical treatment for FIF at our hospital from January 2021 to December 2024 were enrolled. After applying the eligibility criteria, 335 patients were included in the analysis. After PSM, 193 patients (75 males [38.9 %] and 118 females [61.1 %]; mean age, 82.90 ± 9.05 years) were included in the analysis. Of these, 49 (25.4 %) had POP, while 144 (74.6 %) did not. The flowchart of patient selection is shown in Fig. 2. The demographic, clinical, and operative characteristics of the cohort before and after PSM are shown in Table 1.

**Table 1:** Patients’ characteristics and incidence of POP in the overall cohort.

Variables	Before PSM	After PSM
	*n* = 335	*n* = 193
Sex, n (%)		
Male	130 (38.8)	75 (38.9)
Female	205 (61.2)	118 (61.1)
Age (years)	79.95 ± 10.92	82.90 ± 9.05
BMI (kg/m^2^)	21.52 ± 3.52	20.77 ± 3.53
Smoking, n (%)	77 (23.0)	44 (22.8)
Comorbidities, n (%)		
Hypertension	181 (53.0)	109 (56.5)
Diabetes mellitus	41 (12.2)	22 (11.4)
Coronary heart disease	53 (15.8)	32 (16.6)
Chronic obstructive pulmonary disease	4 (1.2)	3 (1.6)
Cerebral infarction	38 (11.3)	25 (13.0)
Duration of surgery (min)	49.00 (37.50, 65.00)	44.00 (35.00, 60.00)
Intraoperative bleeding volume (mL)	200.00 (100.00, 300.00)	200.00 (100.00, 300.00)
Station, n (%)		
Left	136 (40.6)	93 (48.2)
Right	199 (59.4)	100 (51.8)
ISS, mean ± standard deviation	9.94 ± 2.55	9.56 ± 1.67
Injury-to-surgery time, n (%)		
≤2 days	136 (40.6)	79 (40.9)
>2 days	199 (59.4)	114 (59.1)
Postoperative hospital stay (days)	12.43 (7.49, 17.61)	12.59 (7.64, 18.39)
Postoperative complications, n (%)		
POP	53 (15.8)	49 (25.4)
Urinary tract infection	30 (9.0)	19 (9.8)
Delirium	8 (2.4)	6 (3.1)
Heart disease	7 (2.1)	3 (1.6)
Incision infection	6 (1.8)	2 (1.0)
Embolism	5 (1.5)	5 (2.6)
Death	5 (1.5)	3 (1.6)
Other	3 (0.9)	2 (1.0)
Preoperative SII	1089.00 (659.03, 1796.76)	1104.50 (684.25, 1736.43)
Preoperative LMR	2.00 (1.40, 2.80)	2.00 (1.44, 2.75)
Preoperative PLR	157.78 (112.40, 249.58)	163.64 (116.88, 252.50)
Preoperative NLR	6.83 (4.59, 10.33)	6.67 (4.62, 10.20)
Postoperative SII	1317.67 (783.11, 2053.00)	1302.38 (765.00, 1974.86)
Postoperative LMR	1.44 (1.00, 2.00)	1.50 (1.00, 2.00)
Postoperative PLR	201.25 (131.55, 284.37)	192.22 (134.29, 281.82)
Postoperative NLR	8.29 (5.59, 12.15)	8.62 (5.50, 12.33)
Preoperative albumin (g/L)	36.20 ± 4.48	35.90 ± 4.47
Preoperative hemoglobin (g/L)	96.42 ± 22.03	94.64 ± 20.94
Postoperative albumin (g/L)	31.41 ± 3.76	31.12 ± 3.63
Postoperative hemoglobin (g/L)	88.09 ± 18.89	85.10 ± 19.72

Data are presented as n (%), mean ± standard deviation, or M (P25, P75).

BMI, body mass index; pSII, preoperative systemic immune–inflammation index; NLR, neutrophil-to-lymphocyte ratio; PLR, platelet-to-lymphocyte ratio; LMR, lymphocyte-to-monocyte ratio; M (P25, P75), median (25th percentile, 75th percentile); ISS, Injury Severity Score; POP, postoperative pneumonia; PSM, propensity score matching.

PSM effectively eliminated confounding factors (Table 2). Compared with controls, the POP group exhibited a significantly higher postoperative SII (1832.00 [1388.00, 3369.67] vs. 1261.76 [936.44, 1893.94], respectively, P < 0.001) and NLR (13.43 [10.85, 16.67] vs. 7.89 [5.32, 11.00], P < 0.001). The preoperative albumin concentration was similar between the two groups (34.84 ± 4.54 vs. 36.26 ± 4.41, P = 0.056). The postoperative hospital stay was longer in the POP group than in the non-POP group (13.40 days vs. 12.58 days, P = 0.387), but the difference was not statistically significant.

**Table 2:** Patients’ characteristics and incidence of POP in the POP and non-POP groups.

Variables	Before PSM			After PSM		
	POP (*n* = 53)	Non-POP (*n* = 282)	P-value	POP (*n* = 49)	Non-POP (*n* = 144)	P-value
Sex, n (%)			0.862			0.745
Male	20 (37.7)	110 (39.0)		20 (40.8)	55 (38.2)	
Female	33 (62.3)	172 (61.0)		29 (59.2)	89 (61.8)	
Age (years)	83.58 ± 9.10	79.27 ± 11.11	0.008	82.96 ± 9.15	82.88 ± 9.05	0.955
BMI (kg/m^2^)	20.46 ± 3.41	21.72 ± 3.51	0.017	20.72 ± 3.41	20.78 ± 3.59	0.911
Smoking, n (%)	12 (22.6)	65 (23.1)	0.984	11 (22.5)	33 (22.9)	0.946
Comorbidities, n (%)	29 (54.7)	180 (63.8)	0.209	27 (55.1)	97 (67.4)	0.122
Hypertension	26 (49.1)	155 (55.0)	0.428	24 (49.0)	85 (59.0)	0.220
Diabetes mellitus	3 (5.7)	38 (13.5)	0.111	3 (6.1)	19 (13.2)	0.178
Coronary heart disease	8 (15.1)	45 (16.0)	0.874	8 (16.3)	24 (16.7)	0.956
Chronic obstructive pulmonary disease	1 (1.9)	3 (1.1)	0.500	1 (2.0)	2 (1.4)	1.000
Cerebral infarction	6 (11.3)	32 (11.4)	0.996	6 (12.2)	19 (13.2)	0.864
Duration of surgery (min)	45.00 (40.00, 55.00)	50.00 (37.00, 69.00)	0.194	45.00 (40.00, 55.00)	43.00 (35.00, 61.25)	0.580
Intraoperative bleeding volume (mL)	200.00 (100.00, 300.00)	200.00 (100.00, 300.00)	0.669	200.00 (100.00, 300.00)	200.00 (100.00, 300.00)	0.813
Station, n (%)			0.705			0.840
Left	25 (47.2)	141 (50.0)		23 (46.9)	70 (48.6)	
Right	28 (52.8)	141 (50.0)		26 (53.1)	74 (51.4)	
ISS, mean ± standard deviation	9.51 ± 1.48	10.02 ± 2.70	0.052	9.53 ± 1.53	9.58 ± 1.72	0.869
Injury-to-surgery time, n (%)			0.443			0.722
≤2 days	19 (35.9)	117 (41.5)		19 (38.8)	60 (41.7)	
>2 days	34 (64.1)	165 (58.5)		30 (61.2)	84 (58.3)	
Postoperative hospital stay (days)	12.43 (8.45, 20.48)	12.41 (7.48, 17.18)	0.411	13.40 (9.41, 21.33)	12.58 (7.62, 17.47)	0.387
Preoperative SII	1262.50 (833.09, 1980.00)	1074.69 (615.75, 1787.87)	0.164	1262.50 (833.45, 1970.00)	1082.10 (629.19, 1683.98)	0.207
Preoperative LMR	1.75 (1.20, 2.75)	2.00 (1.50, 2.80)	0.198	1.86 (1.33, 2.80)	2.00 (1.57, 2.69)	0.340
Preoperative PLR	180.00 (134.29, 261.25)	155.56 (108.75, 246.07)	0.100	178.57 (133.64, 261.25)	159.49 (111.03, 248.13)	0.249
Preoperative NLR	6.57 (5.57, 11.60)	6.86 (4.46, 10.22)	0.612	6.57 (5.50, 10.00)	6.75 (4.60, 10.26)	0.961
Postoperative SII	1632.00 (1003.00, 3169.67)	1240.18 (779.91, 1971.87)	0.015	1832.00 (1388.00, 3369.67)	1261.76 (936.44, 1893.94)	<0.001
Postoperative LMR	1.20 (1.00, 1.83)	1.50 (1.00, 2.00)	0.100	1.20 (1.00, 1.86)	1.50 (1.00, 2.13)	0.144
Postoperative PLR	235.00 (155.00, 340.00)	197.32 (131.15, 280.00)	0.119	234.29 (155.00, 285.71)	191.12 (131.22, 277.62)	0.235
Postoperative NLR	10.25 (6.22, 13.20)	7.95 (5.51, 11.72)	0.008	13.43 (8.85, 16.67)	7.89 (5.32, 11.00)	<0.001
Preoperative albumin (g/L)	34.93 ± 4.41	36.43 ± 4.46	0.025	34.84 ± 4.54	36.26 ± 4.41	0.056
Preoperative hemoglobin (g/L)	94.08 ± 18.11	96.86 ± 22.69	0.399	94.57 ± 18.65	94.66 ± 21.73	0.979
Postoperative albumin (g/L)	30.37 ± 3.40	31.61 ± 3.80	0.027	30.46 ± 3.38	31.34 ± 3.70	0.141
Postoperative hemoglobin (g/L)	86.13 ± 21.99	88.46 ± 18.27	0.411	85.31 ± 22.40	85.03 ± 18.80	0.932

Data are presented as n (%), mean ± standard deviation, or M (P25, P75).

BMI, body mass index; pSII, preoperative systemic immune–inflammation index; NLR, neutrophil-to-lymphocyte ratio; PLR, platelet-to-lymphocyte ratio; LMR, lymphocyte-to-monocyte ratio; M (P25, P75), median (25th percentile, 75th percentile); ISS, Injury Severity Score; POP, postoperative pneumonia; PSM, propensity score matching.

### 3.2 Risk factors for POP

After PSM, the clinical data were analyzed by univariate and multivariate logistic regression analyses. The univariate analysis revealed that the postoperative SII and NLR were significant risk factors for POP (both P < 0.001). The multivariate analysis showed that postoperative NLR (Exponential(B) [Exp(B)] = 1.279, 95 % confidence interval [CI] 1.083–1.524, P = 0.005) was a significant predictor of POP (Table 3, Table 4).

**Table 3:** Results of the univariate logistic regression analysis for POP.

Variables	B	SE	Wald	P	Exp(B)	95 % Exp(B) CI	
						Down	Up
Sex							
Male	Ref						
Female	−0.11	0.34	−0.325	0.745	0.90	0.46	1.75
Age	0.00	0.02	0.056	0.955	1.00	0.97	1.04
BMI	−0.01	0.05	−0.112	0.911	0.99	0.91	1.09
Smoking	−0.03	0.40	−0.067	0.946	0.97	0.43	2.07
Comorbidities	−0.52	0.34	−1.539	0.124	0.59	0.31	1.16
Hypertension	−0.41	0.33	−1.222	0.222	0.67	0.35	1.28
Diabetes mellitus	−0.85	0.64	−1.312	0.189	0.43	0.10	1.33
Coronary heart disease	−0.02	0.45	−0.055	0.956	0.98	0.39	2.26
Chronic obstructive pulmonary disease	0.39	1.24	0.317	0.751	1.48	0.07	15.77
Cerebral infarction	−0.09	0.50	−0.171	0.864	0.92	0.32	2.33
Duration of surgery	0.00	0.01	0.114	0.909	1.00	0.99	1.01
Intraoperative bleeding volume	−0.00	0.00	−0.511	0.609	1.00	1.00	1.00
Station							
Left	Ref						
Right	0.07	0.33	0.202	0.840	1.07	0.56	2.06
ISS	−0.02	0.10	−0.166	0.868	0.98	0.78	1.18
Injury-to-surgery time							
≤2 days	Ref						
>2 days	0.07	0.33	0.202	0.840	1.07	0.56	2.06
Postoperative hospital stay							
Preoperative SII	0.00	0.00	1.178	0.239	1.00	1.00	1.00
Preoperative LMR	0.01	0.11	0.076	0.939	1.01	0.79	1.25
Preoperative PLR	0.00	0.00	0.652	0.514	1.00	1.00	1.00
Preoperative NLR	0.01	0.03	0.552	0.581	1.01	0.96	1.07
Postoperative SII	0.00	0.00	3.993	<0.001	1.00	1.00	1.00
Postoperative LMR	−0.22	0.20	−1.141	0.254	0.80	0.53	1.14
Postoperative PLR	0.00	0.00	1.139	0.255	1.00	1.00	1.00
Postoperative NLR	0.32	0.06	5.399	<0.001	1.37	1.24	1.56
Preoperative albumin	−0.07	0.04	−1.891	0.059	0.93	0.86	1.00
Preoperative hemoglobin	−0.00	0.01	−0.026	0.979	1.00	0.98	1.02
Postoperative albumin	−0.07	0.05	−1.468	0.142	0.93	0.85	1.02
Postoperative hemoglobin	0.00	0.01	0.086	0.931	1.00	0.98	1.02

BMI, body mass index; pSII, preoperative systemic immune–inflammation index; NLR, neutrophil-to-lymphocyte ratio; PLR, platelet-to-lymphocyte ratio; LMR, lymphocyte-to-monocyte ratio; M (P25, P75), median (25th percentile, 75th percentile); ISS, Injury Severity Score; POP, postoperative pneumonia.

**Table 4:** Results of the multivariate logistic regression analyses for POP.

						95 % Exp(B) CI	
Variables	B	SE	Wald	P	Exp(B)	Down	Up
Postoperative SII	0.00	0.00	0.937	0.349	1.00	1.00	1.00
Postoperative NLR	0.246	0.087	2.836	0.005	1.279	1.083	1.524

CI, confidence interval; SII, preoperative systemic immune–inflammation index; NLR, neutrophil-to-lymphocyte ratio; POP, postoperative pneumonia.

### 3.3 Model calibration

The Hosmer-Lemeshow test was performed to assess the calibration of the logistic regression model. The test resulted in a P-value of 0.191, indicating good agreement between the observed outcomes and the model predictions, and thus confirming adequate model calibration.

### 3.4 Effectiveness of inflammatory markers for predicting POP

**Fig. 3:**
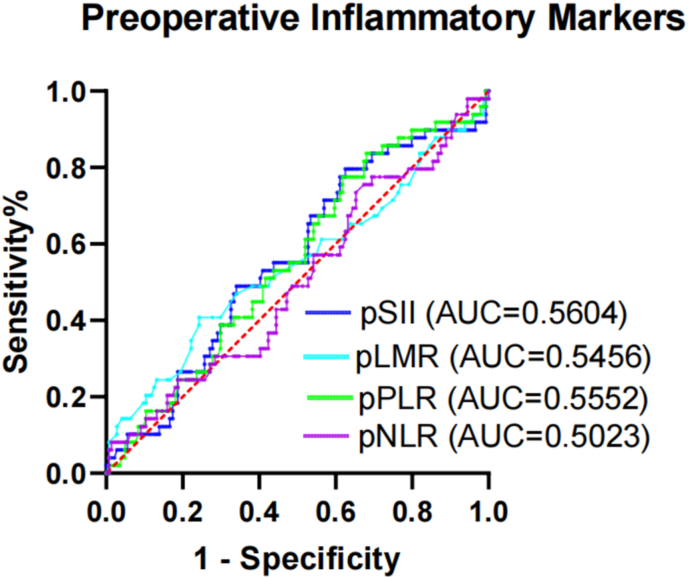
ROC curve analysis of the efficacy of preoperative inflammatory markers for predicting the occurrence of POP in patients with FIF. AUC, area under the curve; pLMR, preoperative lymphocyte-to-monocyte ratio; pNLR, preoperative neutrophil-to-lymphocyte ratio; pPLR, preoperative platelet-to-lymphocyte ratio; POP, postoperative pneumonia; ROC, receiver operating characteristic; pSII, preoperative systemic immune–inflammation index; FIF, femoral intertrochanteric fracture.

**Fig. 4:**
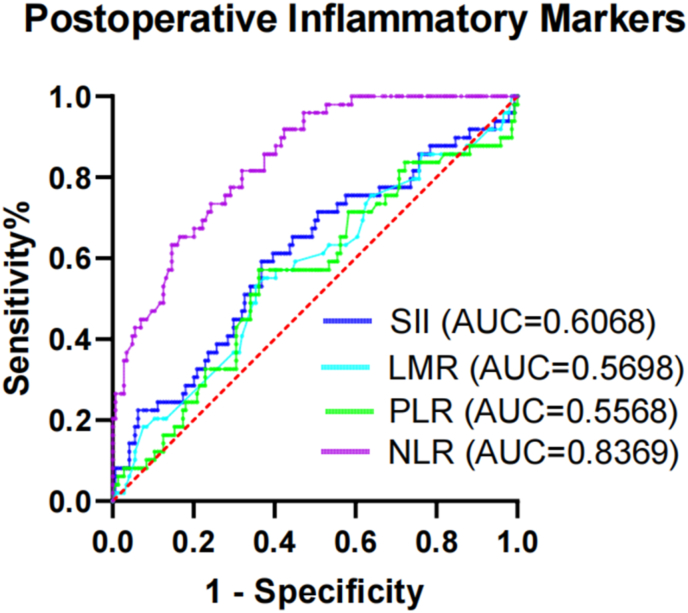
ROC curve analysis of the efficacy of postoperative inflammatory markers for predicting the occurrence of POP in patients with FIF. AUC, area under the curve; LMR, lymphocyte-to-monocyte ratio; NLR, preoperative ratio; POP, postoperative pneumonia; ROC, receiver operating characteristic; SII, systemic immune–inflammation index; FIF, femoral intertrochanteric fracture.

Among the preoperative and postoperative inflammatory markers we analyzed — SII, LMR, PLR, and NLR — the area under the curve (AUC) analysis showed that only postoperative NLR had a strong ability to predict POP (AUC 0.8396, P < 0.001) (Fig. 3, Fig. 4). The optimal threshold for postoperative NLR was determined to be 9.2. The Youden index, sensitivity, and specificity for postoperative NLR were 0.671, 93.02 %, and 74.12 %, respectively.

## 4 Discussion

This study highlights a significant association between postoperative inflammatory dysregulation and the development of postoperative pneumonia (POP) in patients undergoing proximal femoral nail antirotation (PFNA) surgery for femoral intertrochanteric fractures (FIF). The primary finding is that neutrophil-to-lymphocyte ratio (NLR) values are elevated in patients with POP compared to those without. Multivariate analysis confirmed that NLR is an independent predictor of POP, demonstrating discriminatory power (area under the curve [AUC]: 0.8396). This suggests that NLR may serve as a valuable biomarker for risk stratification in this patient population. However, further research is needed to establish the full extent of its clinical utility and to define specific interventions based on NLR values.

Peripheral blood inflammatory markers, such as neutrophils, lymphocytes, macrophages, platelets, and natural killer cells, serve as indicators of systemic inflammation and play diverse roles in cancer progression. ^25^^,^^26^ During the immediate postoperative period, a reduction in the levels and function of lymphocytes and NK cells may compromise cellular immunity, thereby escalating the risk of postoperative pneumonia and other inflammatory conditions. ^27^ In clinical settings, fluctuations in white blood cell counts can influence ratios such as the NLR, lymphocyte-to-monocyte ratio (LMR), systemic immune-inflammation index (SII), and platelet-to-lymphocyte ratio (PLR). These indices are capable of reflecting both systemic and local inflammatory states and can serve as predictive markers for the development of postoperative complications. ^17^^,^^18^^,^^28^^,^^29^

NLR serves as a clinical inflammatory marker, reflecting the balance between neutrophils and lymphocytes, which have distinct roles in the immune system. It indicates potential immunodeficiency, often presenting as lymphocytopenia and neutrophilia. ^30^ Lymphocytes, core immune regulators, may trigger inflammatory cytokine and chemokine cascades under inflammation or stress, promoting neutrophil and macrophage accumulation. ^31^ An elevated NLR may signify abnormal neutrophil - immune cell interactions, disrupting immune responses and increasing the risk of POP. ^32^^,^^33^

A meta-analysis ^17^ demonstrated that elevated preoperative and postoperative NLR correlated with higher long-term mortality risk after surgery for hip fracture in the older population, highlighting the prognostic value of the NLR for survival outcomes. Additionally, multiple studies have linked certain inflammatory markers to postoperative deep vein thrombosis in orthopedic patients. ^28^^,^^29^ Another investigation focusing on POP after surgery for hip fracture identified the NLR as the most reliable predictor of POP in older patients. ^18^ In this study, to rule out the effects of different hip fracture sites and surgical methods, we only studied patients with FIF treated by PFNA. Our results show postoperative NLR has high value in POP prediction (AUC = 0.8369, 95 % CI 1.083–1.524). When postoperative NLR exceeds the optimal threshold of 9.2, there's a statistically significant link between NLR increase and heightened POP susceptibility, which further proves NLR's clinical significance as a POP predictor. However, to our knowledge, no prior studies have specifically explored the relationship between inflammatory markers and POP after PFNA surgery for FIF.

Elevated NLR may guide clinical decision-making in patients at risk of POP. Patients with high NLR could be monitored more closely for respiratory symptoms, enabling earlier detection and intervention. High NLR might also prompt consideration of extended antibiotic prophylaxis or preemptive respiratory support. Recent studies indicate that patients with elevated NLR may benefit from targeted interventions to reduce infection risk. ^34^^,^^35^ In patients with extremely high NLR levels, clinicians might opt to postpone surgery to allow for preoperative optimization, such as initiating anti-inflammatory treatment or enhancing nutritional support.^36^, ^37^, ^38^ However, further research is needed to define the specific clinical actions triggered by elevated NLR and to effectively integrate NLR into clinical pathways.

The predictive power of inflammatory markers for POP in surgical or trauma patients is still unclear. A previous study ^39^ found that PLR and LMR can accurately predict POP in NSCLC surgical patients. However, these ratios may not strongly predict radiographic outcomes one month after surgical resection. Additionally, two other studies showed that SII is a risk factor for postoperative sepsis after bowel obstruction surgery and pulmonary complications after lung cancer resection. ^18^^,^^24^ Surgical trauma can trigger a hyperinflammatory response dominated by neutrophils and suppress lymphocyte-mediated immunoregulation, creating a “double hit” that increases pulmonary infection risk. ^27^^,^^40^^,^^41^ In our study, preoperative and postoperative SII, LMR, and PLR showed no significant link to POP after PFNA surgery for FIF. These findings highlight the varied effects of pre-and postoperative inflammation on POP across different studies, underscoring the need to further clarify this relationship.

Surgical procedures and patients' baseline health status are key factors in POP development. Recent studies have revealed that hip fracture-induced plasma mitochondrial DNA (mtDNA) release can activate the Toll-like receptor 9/nuclear factor-κB pathway, thereby triggering systemic inflammation and lung injury. ^42^^,^^43^ Furthermore, intramedullary nailing procedures may accelerate mtDNA release, potentially exacerbating lung injury in patients with hip fractures and increasing the risk of postoperative pulmonary infection and mortality. ^24^ However, further research is needed to confirm these mechanisms in the context of PFNA surgery and POP.

Previous studies have identified associations between POP risk and factors such as advanced age, low BMI, malnutrition, and high Charlson Comorbidity Index in hip fracture patients. ^44^^,^^45^ However, our PSM analysis did not find significant associations between these factors and POP incidence. This discrepancy may stem from variations in study populations or methodologies. Early mobilization has been shown to reduce the risk of lung infection by 32 % within 24 h postoperatively, ^46^ and predictors like male sex and hypoalbuminemia have also been identified in prior research. ^19^^,^^46^, ^47^, ^48^, ^49^ Nevertheless, our study did not replicate these associations, highlighting the potential influence of context or other variables.

In another study investigating POP following surgery for femoral fracture, 636 patients (mean age: 79.6 ± 8.6 years, 47.8 % male) were included. POP developed in 10.8 % of the patients, with hypertension (78.3 %), diabetes mellitus (60.9 %), and cardiovascular diseases (40.6 %) being the most common comorbidities. The study emphasized prolonged hospitalization and intensive care unit admission as critical consequences of POP, underscoring its socioeconomic burden beyond clinical morbidity. ^50^

Furthermore, another study ^51^ highlighted that delayed surgery (>3 days) was independently associated with an increased risk of POP, emphasizing the importance of timely surgical intervention. Similarly, Bohl et al. ^16^ advocated for evidence-based pneumonia prevention programs targeting high-risk patients, such as males, those aged ≥90 years, those with a BMI of <18.5 kg/m^2^, or patients with COPD. Our findings, while not identifying these factors as significant in the PSM cohort, do not negate their potential importance in the broader, unselected population. The lack of an association in our study could be attributable to homogenization of the cohort after PSM, which may have attenuated the effects of these variables. Therefore, while our results suggest that inflammatory markers like NLR are predictors of POP, they also underscore the need for further research to clarify the roles of surgical timing, patient demographics, and comorbidities in the pathogenesis of POP, particularly in diverse clinical settings. Such investigations would contribute to the development of more comprehensive and personalized prevention strategies for this serious complication.

This study has several limitations. First, its retrospective, single-center design may lead to selection bias and limits generalizability. Second, data from clinical records might affect collection reliability. Though PSM reduced bias, sample size and potential biases could still impact result credibility. Third, the analysis didn't cover inflammatory markers' long-term survival effects. Lastly, sample size constraints prevented differentiation of fracture subtypes and surgical technique variations. Future large-scale, multicenter, prospective studies are needed to confirm the reliability and clinical significance of preoperative inflammatory markers in predicting POP in PFNA patients.

Despite these limitations, this study represents the first evaluation of preoperative and postoperative inflammatory markers as predictors of POP risk following PFNA surgery for FIF. The use of PSM mitigated the confounding biases that are inherent to retrospective studies, strengthening the validity of the associations between inflammatory markers and POP.

In conclusion, this study highlights postoperative NLR as a significant predictor of POP in patients with FIF undergoing PFNA surgery, supporting its clinical utility for risk stratification and early intervention. Further research is warranted to validate these findings and explore the underlying mechanisms.

## CRediT authorship contribution statement

**Fang Li:** Conceptualization, Methodology, Investigation, Data curation, Writing – original draft, Writing – review & editing. **Xiaojun Fu:** Investigation, Data curation, Writing – original draft. **Yingding Ruan:** Methodology, Investigation, Data curation. **Juncheng Yu:** Methodology, Investigation, Data curation. **Junhua Chen:** Methodology, Investigation, Data curation. **Liming Xu:** Methodology, Investigation, Data curation. **Jie Xiao:** Conceptualization, Supervision, Writing – review & editing.

## Consent for publication

Not applicable.

## Ethical approval

The research was ethically implemented in strict adherence to the Declaration of Helsinki principles, with formal approval granted by the Institutional Ethics Committee of The First People's Hospital of Jiande (Ethics Committee Approval Number: 20250613-KY-001). In alignment with the study's retrospective methodology, the ethics committee formally dispensed with the requirement for written informed consent.

## Clinical Trial number

Not applicable.

## AI and AI-assisted technologies

In the preparation of this work, the authors did not utilize any AI technology to edit the manuscript. The authors take full responsibility for the content of the publication.

## Funding

This research was supported by the Jiande Municipal Science and Technology Bureau (Grant No. 2024YW06).
